# Effects of litter exposure and flock age of broiler breeders on hatchability and the microbial composition of eggshells, egg membranes, and egg contents

**DOI:** 10.3389/fvets.2025.1589607

**Published:** 2025-06-26

**Authors:** Gábor Csitári, Nikoletta Such, László Menyhárt, Kornél Schermann, Kornél Humpok, Valéria Farkas, László Pál, Károly Dublecz

**Affiliations:** ^1^Department of Animal Nutrition and Nutritional Physiology, Institute of Physiology and Nutrition, Georgikon Campus, Hungarian University of Agriculture and Life Sciences, Keszthely, Hungary; ^2^Department of Applied Statistics, Institute of Mathematics and Basic Sciences, Budai Campus, Hungarian University of Agriculture and Life Sciences, Budapest, Hungary; ^3^Gallus Ltd. of Poultry Breeding and Hatching, Devecser, Hungary

**Keywords:** floor egg, breeders’ age, broiler breeders, hatchability, microbiome, eggshell, egg membrane, egg content

## Abstract

Floor eggs represent a significant loss for broiler breeder farms and hatcheries due to the increased risk of bacterial contamination of embryos, the hatchery environment, and newly hatched chicks. In this trial, the effects of litter exposure duration (3, 6, and 16 h) and breeder flock age (22–23 weeks vs. 65–66 weeks) were evaluated in terms of hatchability and the microbial composition of different egg components (eggshell, egg membrane, and egg content). The number of total culturable aerobic microbes on the eggshell surface increased following litter exposure and decreased after 3 h. Hatchability, however, was significantly reduced only after 16 h of exposure, primarily due to increased embryonic mortality. Litter exposure and flock age led to significant differences in microbiota composition, but only on the eggshell surface. At the phylum level, 16 h of litter exposure significantly increased the abundance of *Firmicutes* and decreased that of *Proteobacteria*. At the genus level, litter exposure resulted in increased relative abundances of *Lactobacillus* and *Ruminococcus torques group* and a decreased abundance of *Staphylococcus*. The microbiota of the egg membrane and egg content were similar at the phylum level; however, notable differences were observed at the genus level. *Pseudomonas* was dominant in the egg membrane but underrepresented in the egg content, leading to a significantly higher abundance of spoilage-associated bacterial genera in the membrane than in the content. Interestingly, the genus *Flexivirga* (phylum *Actinobacteria*) was detected in high abundance in both the egg membrane and egg content, despite not having been previously reported inside eggs. According to the results, no measurable bacterial translocation from the litter into the internal egg structures was observed. However, the frequency of floor egg collection may represent a critical factor if such eggs are intended for hatching.

## 1 Introduction

In hatching egg production, floor eggs represent a significant source of loss for broiler breeder farms and pose considerable risks of bacterial contamination within the hatchery. A floor egg refers to an egg laid outside the designated nest area, typically in manure. Several recent studies have confirmed that floor eggs generally exhibit reduced hatchability compared to conventional nest eggs (1–3). However, in some cases, increased microbial contamination has not resulted in lower hatchability (4), and the eggshell microbiota may even serve as a protective barrier against pathogens (5).

The eggshell microbiota typically comprises microorganisms originating from both the hen and the surrounding litter (6). Commonly represented bacterial phyla on the eggshell include *Firmicutes*, *Bacteroidetes*, and *Actinobacteria*, while *Proteobacteria* are often underrepresented. Dominant genera include *Bacteroides*, *Blautia*, *Clostridium*, *Faecalibacterium*, *Lactobacillus*, *Megamonas*, and *Oscillibacter* (5–9).

Poultry litter is a complex matrix comprising bedding materials, chicken excreta, spoiled feed, feathers, and other environmental elements (10–12). It harbors a dynamic microbiome influenced by various factors, such as the animals’ gut microbiota, manure dry matter content, ventilation, and environmental temperature and humidity (10, 13, 14). Litter amendments, such as acidifiers, alkalizers, charcoal, and gypsum, can also modify the microbiome (15). Several studies have explored interactions between litter and the gastrointestinal microbiome, as well as the occurrence of pathogens (14, 16). This interaction is relevant in both broilers and layers, as the gut microbiota can affect meat and egg safety. Ingestion of manure by young chicks is considered to play a key role in the development of the gut microbiota (10). Despite ongoing research, the interaction between the litter microbiome and the host remains largely unexplored (17).

Egg spoilage can result from microbial contamination, particularly when bacteria penetrate the eggshell and proliferate in the albumen or yolk. Spoilage-associated genera reported in the literature include *Alcaligenes*, *Enterococcus*, *Escherichia/Shigella*, *Proteus*, *Pseudomonas*, *Staphylococcus*, and *Streptococcus* (18–20). Freshly laid eggs are particularly vulnerable due to their moist surface. Although eggs possess natural defense mechanisms, Gram-negative bacteria such as *Pseudomonas*, *Alcaligenes*, and *Salmonella* are among the most common invaders (21). To prevent trans-shell bacterial penetration, hatching eggs are typically sanitized, significantly reducing microbial loads (4, 7, 22). Post-sanitization, the remaining eggshell microbiota has minimal impact on the developing embryo. However, some spore-forming bacteria, resistant to disinfection and oxygen stress, may survive and colonize chicks during hatching (7, 23).

Lower hatchability of floor eggs may also result from prolonged contact with unsuitable litter conditions. These eggs are typically collected after a delay and cooled later, exposing them to inappropriate temperature and moisture, which negatively affects quality. Hen age is another important factor influencing egg weight and hatchability (24, 25). Older hens tend to lay larger eggs with thinner shells, and their eggs often exhibit reduced hatchability (26–28). However, some studies report conflicting results; for example, Roque and Soares (29) found that hatchability and viability were lower in younger flocks due to increased early and late embryonic mortality. Pre-hatch storage time and environmental temperature also impair hatchability in an age-dependent manner (30, 31).

Eggshell microbial integrity is similarly influenced by hen age. As shell quality declines with age, the risk of microbial penetration increases (32). The total bacterial load on the eggshell surface has been shown to increase with flock age (33). Moreover, the intestinal microbiota of layer hens has been experimentally associated with egg quality (34). Despite controlling for breed, age, and environment, large variations in hatchability between flocks are frequently observed (35), suggesting the existence of other, currently unknown influencing factors.

Microorganisms can also enter the egg internally via the hen’s reproductive tract (36, 37), which is not sterile (37–39) and shares similarities with the gut microbiome (39). Dominant phyla in the reproductive tract include *Proteobacteria*, *Firmicutes*, *Actinobacteria*, and *Bacteroidetes*, with their abundances influenced by factors such as growth rate (37, 38, 40–42). It is hypothesized that bacteria from the reproductive tract may translocate into the forming egg, becoming localized between the chorion and inner membrane or even within the developing embryo (37, 43).

The present study aimed to investigate whether the time eggs spend in the litter and the age of breeder flocks influence hatchability and the microbial composition of various egg components (eggshell, inner membrane, and egg content). Special attention was given to the inner egg membrane, as it may serve as the first barrier to bacterial invasion through the shell. Our findings aim to provide practical insights for broiler breeder farms and hatcheries regarding the risks and viability of using floor eggs for hatching.

## 2 Materials and methods

### 2.1 Egg collection and hatching

A total of 1,440 hatching eggs (720 eggs per flock) were collected from two Ross 308 parent flocks of different ages—22–23 weeks and 65–66 weeks—from separate farms operated by Gallus Ltd. (Bakonypölöske and Oroszi, Hungary). Eggs were transported to the Gallus Ltd. hatchery in Devecser, Hungary, using air-conditioned vehicles to maintain egg quality during transit.

To simulate floor egg conditions and assess the impact of litter exposure duration, four treatment groups were established: Control group: Conventional nest eggs collected from the collecting belt during the second egg collection of the day; these eggs were freshly laid and not contaminated with litter. Litter treatment groups: Eggs subjected to 3-, 6-, or 16-h exposure to poultry litter. The eggs used for litter treatments were originally nest eggs and manually placed in litter within different areas of the barn. Each egg was gently mixed with the litter once per hour using sterile gloves to mimic natural floor egg conditions. To prevent hen contact, plastic baskets were placed over the eggs. After the designated exposure periods (3, 6, or 16 h), eggs were collected and stored at 16–18°C and 75–80% relative humidity until hatching.

The distribution of eggs across treatment groups was as follows: control and 16-h treatment: 185 eggs each, 3-h and 6-h treatments: 180 eggs each. The discrepancy in sample size reflects the design of the microbiota analysis: only the control and 16-h groups were subjected to next-generation sequencing (NGS). For microbiological analysis, five eggs from each treatment group (per farm) were transported to the laboratory for culturable microbiota assessment. An additional five eggs from the control and 16-h groups (per farm) were sent for NGS-based microbial community analysis, focusing on the eggshell, inner membrane, and egg content. The remaining 175 eggs per treatment group were incubated at the Gallus Ltd. hatchery. All eggs were incubated under identical conditions according to the standard procedures of the hatchery. Prior to incubation, eggs were sanitized using formaldehyde fumigation at a concentration of 7 g/m^3^ paraformaldehyde.

Hatching commenced on day 7 post-laying in Petersime BioStreamer 24S pre-hatching machines. Environmental parameters, including temperature, relative humidity, CO₂ concentration, and egg turning, were automatically regulated based on the manufacturer’s standard hatching program.

On day 18 of incubation, candling was performed to identify and remove infertile, dead, damaged, or rotten eggs. Fertile eggs were then vaccinated in ovo against infectious bursal disease (IBD) using an automated injection system (Embrex Inovoject, Zoetis Inc., New York, United States). Post-vaccination, eggs were automatically transferred to hatcher trays, and incubation continued under controlled conditions. Hatchability was calculated based on both total and fertile eggs. Embryonic mortality was expressed as a percentage of fertile eggs.

### 2.2 Microbiological evaluation of eggshells by culturing

Microbiological analysis of the eggshells was conducted at the Microbiological Laboratory of Gallus Ltd. (Devecser, Hungary). A total of 40 eggs were analyzed, comprising 20 eggs from the younger flock and 20 from the older flock. Each litter treatment group (0, 3, 6, and 16 h) included five replicate eggs per age group. All microbiological assessments were performed prior to hatchery sanitization.

Sample preparation followed the ISO 6887-4:2017 standard. The following parameters were assessed: total aerobic bacterial counts, determined using ISO 4833-1:2014, and coliform counts, determined using ISO 21528-2:2017. For culturing, plate count agar (PCA) was used for total aerobic bacteria, while violet Red Bile Glucose (VRBG) agar was used for coliform enumeration.

### 2.3 DNA sequencing and microbial community analysis

For DNA sequencing, a total of 20 eggs were collected, 10 from the younger and 10 from the older flock. Only two treatment groups were analyzed: control nest eggs and eggs exposed to 16-h litter treatment, each in five replicates per age group.

#### 2.3.1 Sample preparation

Eggshells were washed using 1.2 mL DNA/RNA Shield solution (Zymo Research, CA, USA), and then transferred into DNA/RNA shield lysis tubes for DNA extraction. For the inner egg membrane, the eggs were washed with detergent, surface-sterilized three times by flaming with ethanol, and then aseptically broken. The inner membrane was collected using a sterile scalpel.

For the egg content, a 150 μL mixture of egg white and yolk was sampled using a sterile pipette.

#### 2.3.2 DNA extraction and quantification

DNA was extracted from the eggshell, egg membrane and egg content samples using the ZymboBIOMICS 96 MagBead DNA Kit with ZR BashingBed Lysis Tubes (Zymo Research, CA, USA). DNA concentration was measured with a Qubit 3.0 Fluorometer using the Qubit dsDNA HS Assay Kit (Thermo Fisher Scientific, Waltham, MA, United States).

#### 2.3.3 Library preparation and sequencing

The bacterial 16S rRNA V3–V4 region was amplified using tagged primers. PCR amplification and library purification followed Illumina’s 16S Metagenomic Sequencing Library Preparation protocol (44). Libraries were validated using High Sensitivity D1000 ScreenTape on a TapeStation 2,200 system (Agilent Technologies, Santa Clara, CA, United States). Equimolar pooled libraries were sequenced on the Illumina MiSeq platform with a MiSeq Reagent Kit v3 (600 cycles) using a 300-bp paired-end read format.

The raw sequence data were deposited in the NCBI Sequence Read Archive (SRA) under BioProject ID PRJNA1012860.

#### 2.3.4 Data analysis

Using next-generation sequencing results, the relative abundance of egg spoilage-associated bacterial genera was calculated. This included the sum of reads assigned to the following genera: *Alcaligenes*, *Enterococcus*, *Escherichia/Shigella*, *Proteus*, *Pseudomonas*, *Staphylococcus*, and *Streptococcus*.

### 2.4 Bioinformatics and statistical analyses

Bacterial communities were identified by analyzing the V3–V4 region of the 16S rRNA gene using the Illumina MiSeq platform. Sequence data were processed using QIIME2 (Quantitative Insights Into Microbial Ecology 2), version 2020.2 (45).

#### 2.4.1 Sequence processing and OTU clustering

Raw sequences were quality-filtered based on Phred quality scores and the presence of ambiguous bases using the quality-filter q-score plugin. Denoising was performed using the Deblur method (deblur denoise-16S), and representative sequences were identified using a 16S reference as a positive filter. Sequences were clustered into operational taxonomic units (OTUs) using the VSEARCH open-reference algorithm, with a 97% similarity threshold against the SILVA reference database (release 132) (46).

#### 2.4.2 Diversity analyses

Alpha diversity metrics—including species richness, Chao1, Shannon, and Simpson indices—were calculated using the qiime2-diversity plugin. Beta diversity was assessed using Bray–Curtis dissimilarity. All diversity analyses were conducted after rarefaction to 1,000 sequences per sample. Supplementary analysis was performed using MicrobiomeAnalyst (47).

#### 2.4.3 Statistical analysis

A two-way ANOVA was conducted to assess the effects of litter treatment and flock age on microbiota culturing and hatchability. A three-way ANOVA was used to analyze OTU numbers, alpha diversity metrics, and the relative abundance of spoilage-associated bacterial genera, with egg part, litter treatment, and flock age as the main factors.

All ANOVA analyses were performed using R software (48).

#### 2.4.4 Taxonomic abundance testing

Relative abundance data at different taxonomic levels were analyzed using the Aligned Rank Transform (ART) for non-parametric factorial analysis, implemented via the ARTool R package (49). This method enables valid testing of main effects and interactions in non-parametric factorial designs by aligning and rank-transforming the data, followed by standard ANOVA on the aligned ranks.

For each taxon, the primary effects of litter treatment, flock age, and their interaction were tested separately for each egg part (eggshell, egg membrane, and egg content). To correct for multiple testing, *p*-values were adjusted using the Benjamini–Hochberg False Discovery Rate (BH-FDR) procedure (50). A BH-adjusted *p*-value < 0.05 was considered statistically significant.

The ART procedure has been validated as a robust and suitable alternative for microbiome data analysis in designs with complex interactions and non-normal distributions (51, 52).

## 3 Results

### 3.1 Egg weight and culturable microbes of the eggshell

The weight of the eggs ranged from 49.2 to 77.0 g, with eggs from the older breeder flock being significantly heavier, as expected (Table 1). The total aerobic bacterial counts on the eggshells increased proportionally with the duration of litter exposure, and all litter-treated groups showed significantly higher counts compared to the control group (Table 1). The age of the breeder flock also influenced bacterial load: eggs from the younger hens exhibited higher aerobic bacterial counts on their shells across all treatments. In contrast, coliform bacteria were not detected on any of the eggshells, remaining below the detection limit of 1 × 10^2^ CFU/egg in all cases.

**Table 1:** The effect of litter treatments and the age of laying hens on the egg weight and the number of culturable microbes of eggshell (*n* = 10).

	Weight of egg (g)	Number of total aerobic bacteria (log_10_ CFU/egg)	Number of coliforms (log_10_ CFU/egg)
Litter treatment (hours spent in litter)			
0	61.8^ab^	4.88^b^	< 2.00
3	62.9^a^	5.87^a^	< 2.00
6	61.0^ab^	5.80^a^	< 2.00
16	59.7^b^	5.73^a^	< 2.00
Age of breeders			
Young (22–23 weeks)	53.2^b^	5.77^a^	< 2.00
Old (65–66 weeks)	69.5^a^	5.38^b^	< 2.00
Pooled SEM*	0.538	0.057	*-*
*p*-values			
Litter treatment	0.041	0.000	Nd
Age of breeders	0.000	0.000	Nd
Litter treatment × Age of breeders	0.288	0.000	nd

nd: not determined; *: SEM: standard error of mean; ^a,b^ means with different superscripts of the same column are significantly different (*p* < 0.05).

### 3.2 Hatching parameters

Fertility rates and the proportion of damaged eggs were not significantly affected by litter treatment duration (Table 2). However, exposure to litter for 16 h resulted in a significant increase in embryonic mortality and a decrease in hatchability compared to the 3- and 6-h litter exposure groups. Additionally, eggs from the younger breeder flock exhibited a significantly higher hatchability rate relative to the total number of incubated eggs (Table 2).

**Table 2:** The effect of litter treatments and the age of laying hens on the hatching parameters (*n* = 175).

	Fertility (% of set)	Damaged (% of set)	Mortality (% of fertile)	Hatchability (% of fertile)	Hatchability (% of set)
Litter treatment (hours spent in litter)					
0	96.3	0.28	5.0^ab^	94.8^ab^	91.3^ab^
3	97.5	0.28	4.16^b^	95.7^a^	93.3^a^
6	96.9	0.28	3.33^b^	96.5^a^	93.6^a^
16	96.5	0.56	8.05^a^	91.5^b^	87.5^b^
Age of breeders					
Young (22–23 weeks)	97.5	0.14	4.44	95.4	92.9^a^
Old (65–66 weeks)	95.6	0.56	5.83	93.8	89.2^b^
Pooled SEM*	1.58	0.29	2.17	2.33	3.30
*p*-values					
Litter treatment	ns	ns	<0.05	<0.05	<0.05
Age of breeders	ns	ns	ns	ns	<0.05
Litter treatment × age of breeders	ns	ns	ns	ns	ns

ns: not significant at a *p*-value of >0.05 level; *: SEM: standard error of mean; ^a,b^ means with different superscripts of the same column are significantly different (*p* < 0.05).

### 3.3 Results of next-generation sequencing

In this study, a total of 1,207,766 good-quality 16S rRNA reads were obtained for analysis from all 40 samples after quality filtering. The overall average sequence numbers were 20,129 (minimum: 10,845; maximum: 31,174). When separated by egg parts, the average sequence numbers were 19,020 (minimum: 15,605; maximum: 26,592) on the eggshell, 19,197 (minimum: 15,151; maximum: 24,610) in the inner membrane, and 22,172 (minimum: 10,845; maximum: 31,174) in the egg content. These sequences were assigned to 2,643 OTUs at 97% similarity using the open approach. A total of 1,498 OTUs remained after the data filtering step in MicobiomeAnalyst.

#### 3.3.1 Alpha and beta diversities

All alpha diversity indices were significantly influenced by the egg component (eggshell, inner membrane, and egg content). The eggshell microbiome exhibited the highest OTU count and Chao1 index, followed by the inner membrane and then the egg content (Table 3). For the Shannon and Simpson indices, the 16-h litter exposure significantly reduced microbial alpha diversity. However, due to significant interactions between the main factors, these effects cannot be interpreted independently. Breeder age had no significant impact on any of the diversity indices.

**Table 3:** Treatment effects on the OTU numbers and bacterial alpha diversity indices (*n* = 15 for litter treatment; *n* = 5).

	OTU	Chao1	Shannon	Simpson
Litter treatment (hours spent in litter)				
0	229	229	3.945^a^	0.946^a^
16	217	217	3.780^b^	0.918^b^
Age of breeders				
Young (22–23 weeks)	232	232	3.914	0.929
Old (65–66 weeks)	214	214	3.811	0.936
Egg parts				
Shell	425^a^	426^a^	3.569^b^	0.868^b^
Membrane	146^b^	146^b^	4.112^a^	0.963^a^
Content	98^c^	98^c^	3.906^a^	0.965^a^
Pooled SEM*	10.15	10.16	0.056	0.006
*p*-values				
Litter treatment	0.308	0.293	0.043	0.002
Age of breeders	0.126	0.126	0.201	0.433
Egg parts	0.001	0.001	0.001	0.001
Litter treatment × age of breeders	0.603	0.593	0.072	0.003
Litter treatment × egg part	0.090	0.087	0.001	0.001
Age of breeders × egg part	0.577	0.581	0.159	0.009
Litter treatment × age of breeders × egg part	0.144	0.150	0.272	0.004

^a,b^ means with different superscripts of the main treatment averages are significantly different (*p* < 0.05).

**Figure 1:**
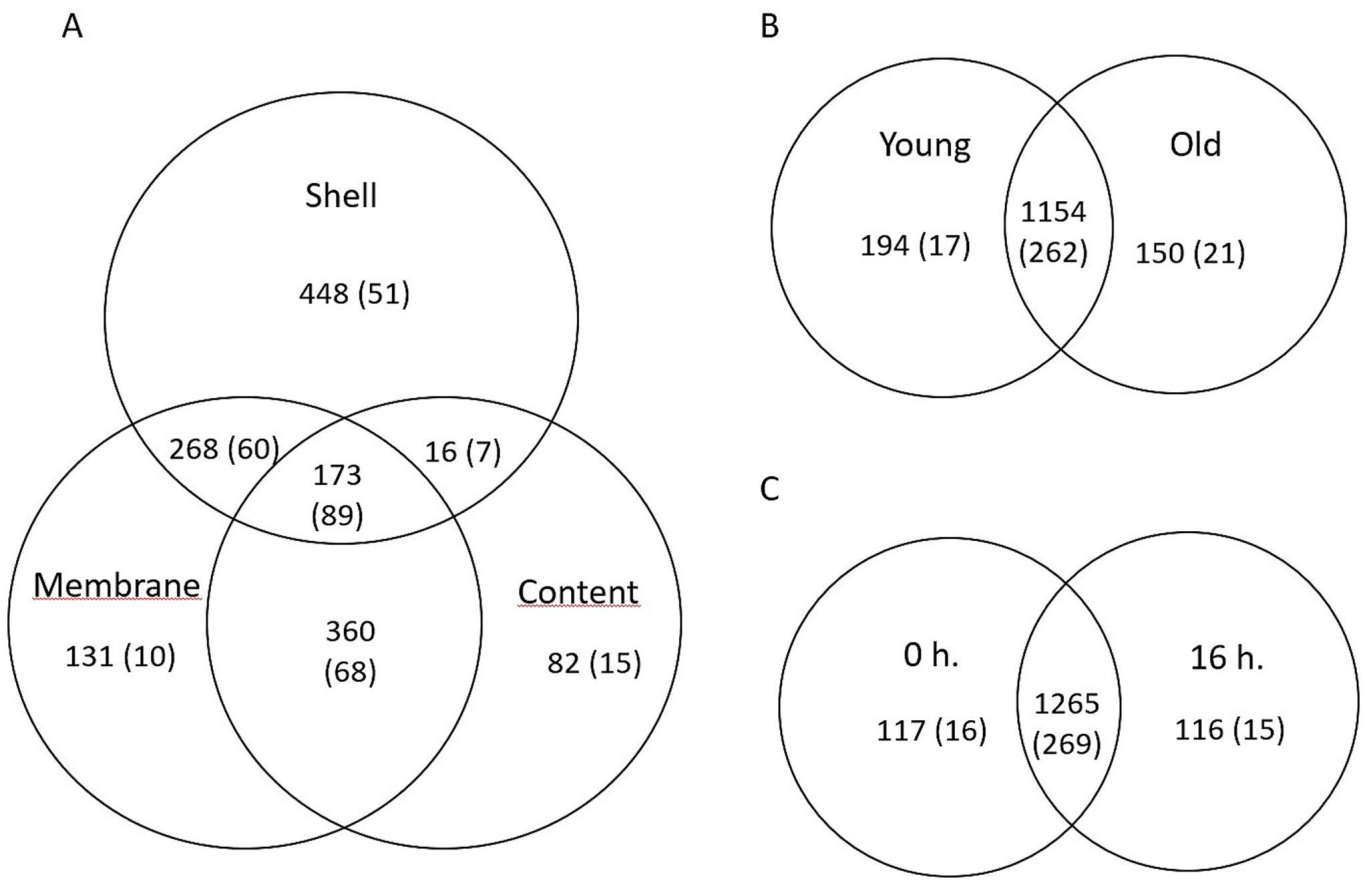
Principal coordinate analysis (PCoA) based on the Bray–Curtis dissimilarity matrix depicting bacterial community composition as influenced by **(A)** egg parts, **(B)** breeder age, and **(C)** litter treatment. Statistical differences in beta diversity were assessed using permutational multivariate analysis of variance (PERMANOVA).

Beta diversity analysis was performed to assess differences in microbial community composition among the samples (Figure 1). Principal coordinate analysis (PCoA) based on the Bray–Curtis dissimilarity matrix revealed significant clustering by egg part (PERMANOVA, R^2^ = 0.39, *p* = 0.001), with the microbial communities of eggshells distinctly separated from those of the inner membrane and egg content (Figure 1A). The inner membrane and egg content microbiota exhibited high similarity. The effect of hen age showed a trend toward separation (R^2^ = 0.03, *p* = 0.058) (Figure 1B), whereas litter treatment had no significant impact on microbial community structure, as indicated by a considerable overlap between litter-treated and untreated eggs (Figure 1C).

1A: [PERMANOVA] F-value: 17.964; R-squared: 0.38662; *p-*value: 0.001.

1B: [PERMANOVA] F-value: 1.8807; R-squared: 0.031407; *p*-value: 0.058.

1C: [PERMANOVA] F-value: 0.77102; R-squared: 0.013119; *p*-value: 0.607.

#### 3.3.2 Taxonomic microbiota composition of eggshell

Four bacterial phyla—*Firmicutes, Bacteroidetes, Actinobacteria*, and *Proteobacteria*—were detected on the eggshell at relative abundances exceeding 1%, with *Firmicutes* being the dominant phylum. The relative abundance of these phyla was significantly influenced by both litter treatment and the age of the hens (Table 4). Specifically, litter treatment increased the abundance of *Firmicutes* and decreased that of *Proteobacteria* on the eggshell compared to nest eggs. Eggs from older flocks exhibited significantly lower frequencies of *Firmicutes* and *Bacteroidetes*, while *Actinobacteria* abundance was approximately two-fold higher compared to eggs from younger flocks. At the genus level, *Staphylococcus, Lactobacillus*, and Salinicoccus were the most abundant on the eggshell surface (Supplementary Table S1). Litter treatment significantly reduced the abundance of *Staphylococcus* and increased that of *Lactobacillus* and the *Ruminococcus_torques_group*. Additionally, breeder flock age affected genus distribution; eggshells from older flocks had significantly lower proportions of *Staphylococcus* and *Lactobacillus*, but higher abundances of *Salinicoccus, Brachybacterium*, and *Brevibacterium*.

**Table 4:** Treatment effects on the relative abundances of bacterial phyla of the eggshell (%) (*n* = 5).

Phylum	Litter treatment	Age of breeders		Mean (litter treatment)	BH-corrected *p*-value		
		Young	Old		Litter treatment	Age of breeders	Interaction
*Firmicutes*	0 h in litter	73.89	67.86	70.87^b^	0.000	0.000	0.204
	16 h in litter	80.58	70.46	75.52^a^			
	mean (age of breeders)	77.24^a^	69.16^b^				
*Bacteroidetes*	0 h in litter	11.05	7.77	9.41	0.238	0.012	1.000
	16 h in litter	9.45	6.21	7.83			
	mean (age of breeders)	10.25^a^	6.99^b^				
*Actinobacteria*	0 h in litter	10.85	21.15	16.00	0.084	0.000	0.026
	16 h in litter	7.36	21.35	14.35			
	mean (age of breeders)	9.10	21.25				
*Proteobacteria*	0 h in litter	1.57	1.34	1.45^a^	0.001	0.352	0.536
	16 h in litter	0.88	0.75	0.81^b^			
	mean (age of breeders)	1.22	1.05				

The table contains only phyla above 1% relative abundance; ^a,b^ means with different superscripts of the main treatment averages are significantly different (*p* < 0.05).

#### 3.3.3 Taxonomic microbiota composition of egg membranes

Eight bacterial phyla were detected at relative abundances exceeding 1% in the egg membranes. Among these phyla, *Proteobacteria* and *Firmicutes* were dominant, while *Actinobacteria*, *Cyanobacteria*, and *Bacteroidetes* each comprised more than 10% of the community (Table 5). No significant effects of litter treatment or breeder age were observed at either the phylum or genus level. The 10 most abundant genera identified in the egg membrane included *Pseudomonas*, *Flexivirga*, *Staphylococcus*, *Paracoccus*, *Rhodanobacter*, *Enhydrobacter*, *Candidimonas*, *Chujaibacter*, *Ruminococcaceae_NK4A214_group*, and *Salinicoccus* (Supplementary Table S2).

**Table 5:** Changes in the relative abundances of bacterial phyla in the egg membrane (%) (*n* = 5).

Phylum	Litter treatment	Age of breeders		Mean (litter treatment)	BH-corrected *p*-value		
		Young	Old		Litter treatment	Age of breeder	Interaction
*Proteobacteria*	0 h in litter	29.91	24.23	27.07	0.757	0.735	0.757
	16 h in litter	29.75	28.95	29.35			
	mean (age of breeders)	29.83	26.59				
*Firmicutes*	0 h in litter	25.05	25.15	25.10	0.757	0.735	0.945
	16 h in litter	23.62	29.65	26.64			
	mean (age of breeders)	24.34	27.40				
*Actinobacteria*	0 h in litter	13.07	12.68	12.88	0.735	0.945	0.738
	16 h in litter	10.12	12.67	11.39			
	mean (age of breeders)	11.60	12.67				
*Cyanobacteria*	0 h in litter	14.24	19.48	16.86	0.735	0.735	0.735
	16 h in litter	11.26	13.91	12.59			
	mean (age of breeders)	12.75	16.70				
*Bacteroidetes*	0 h in litter	5.72	6.09	5.91	0.735	0.735	0.735
	16 h in litter	10.33	4.88	7.61			
	mean (age of breeders)	8.03	5.49				
*Patescibacteria*	0 h in litter	1.46	1.93	1.69	0.735	0.735	0.735
	16 h in litter	2.16	1.21	1.69			
	mean (age of breeders)	1.81	1.57				
*Chlamydiae*	0 h in litter	3.66	2.68	3.17	0.735	0.735	0.945
	16 h in litter	2.80	1.72	2.26			
	mean (age of breeders)	3.23	2.20				
*Planctomycetes*	0 h in litter	0.82	0.90	0.86	0.757	0.945	0.735
	16 h in litter	1.16	1.22	1.19			
	mean (age of breeders)	0.99	1.06				

The table contains only phyla above 1% relative abundance.

#### 3.3.4 Taxonomic microbiota composition of egg contents

The egg content, comprising a mixture of egg yolk and egg white, comprised eight bacterial phyla with relative abundances above 1% (Table 6). Alongside the dominant *Proteobacteria* and *Firmicutes, Actinobacteria* and *Cyanobacteria* were present at frequencies exceeding 10%. Similar to the egg membrane, no significant differences were observed at any taxonomic level. The 10 most abundant genera in the egg content, based on relative abundance, were *Flexivirga* (9.39%), *Rhodanobacter* (3.90%), *Paracoccus* (3.17%), *Rhodococcus* (2.95%), *Chujaibacter* (2.62%), *Alkanibacter* (2.37%), *Mycobacterium* (1.97%), *Ruminococcaceae_NK4A214_group* (1.86%), *Enhydrobacter* (1.80%), and *Lactobacillus* (1.58%) (Supplementary Table S3).

**Table 6:** Changes in the relative abundances of bacterial phyla in the egg content (%) (*n* = 5).

Phylum	Litter treatment	Age of breeders		Mean (litter treatment)	BH-corrected *p*-value		
		Young	Old		Litter treatment	Age of breeder	Interaction
*Proteobacteria*	0 h in litter	29.15	27.33	28.24	1.000	1.000	1.000
	16 h in litter	31.90	22.24	27.07			
	mean (age of breeders)	30.53	24.79				
*Firmicutes*	0 h in litter	21.58	19.60	20.59	1.000	1.000	1.000
	16 h in litter	20.44	28.37	24.40			
	mean (age of breeders)	21.01	23.99				
*Actinobacteria*	0 h in litter	13.99	15.59	14.79	1.000	1.000	1.000
	16 h in litter	13.61	12.96	13.29			
	mean (age of breeders)	13.80	14.28				
*Cyanobacteria*	0 h in litter	14.30	14.05	14.18	1.000	1.000	1.000
	16 h in litter	12.55	14.12	13.33			
	mean (age of breeders)	13.42	14.08				
*Bacteroidetes*	0 h in litter	4.30	9.08	6.69	1.000	1.000	1.000
	16 h in litter	5.31	7.82	6.56			
	mean (age of breeders)	4.80	8.45				
*Planctomycetes*	0 h in litter	0.73	1.34	1.03	1.000	1.000	1.000
	16 h in litter	1.68	0.67	1.17			
	mean (age of breeders)	1.20	1.00				
*Chlamydiae*	0 h in litter	3.51	1.81	2.66	1.000	1.000	1.000
	16 h in litter	1.66	2.86	2.26			
	mean (age of breeders)	2.59	2.34				
*Verrucomicrobia*	0 h in litter	2.30	2.70	2.50	1.000	1.000	1.000
	16 h in litter	3.09	2.58	2.83			
	mean (age of breeders)	2.69	2.64				

The table contains only phyla above 1% relative abundance.

#### 3.3.5 Common OTUs among eggshell, egg membrane, and egg contents

**Figure 2:**
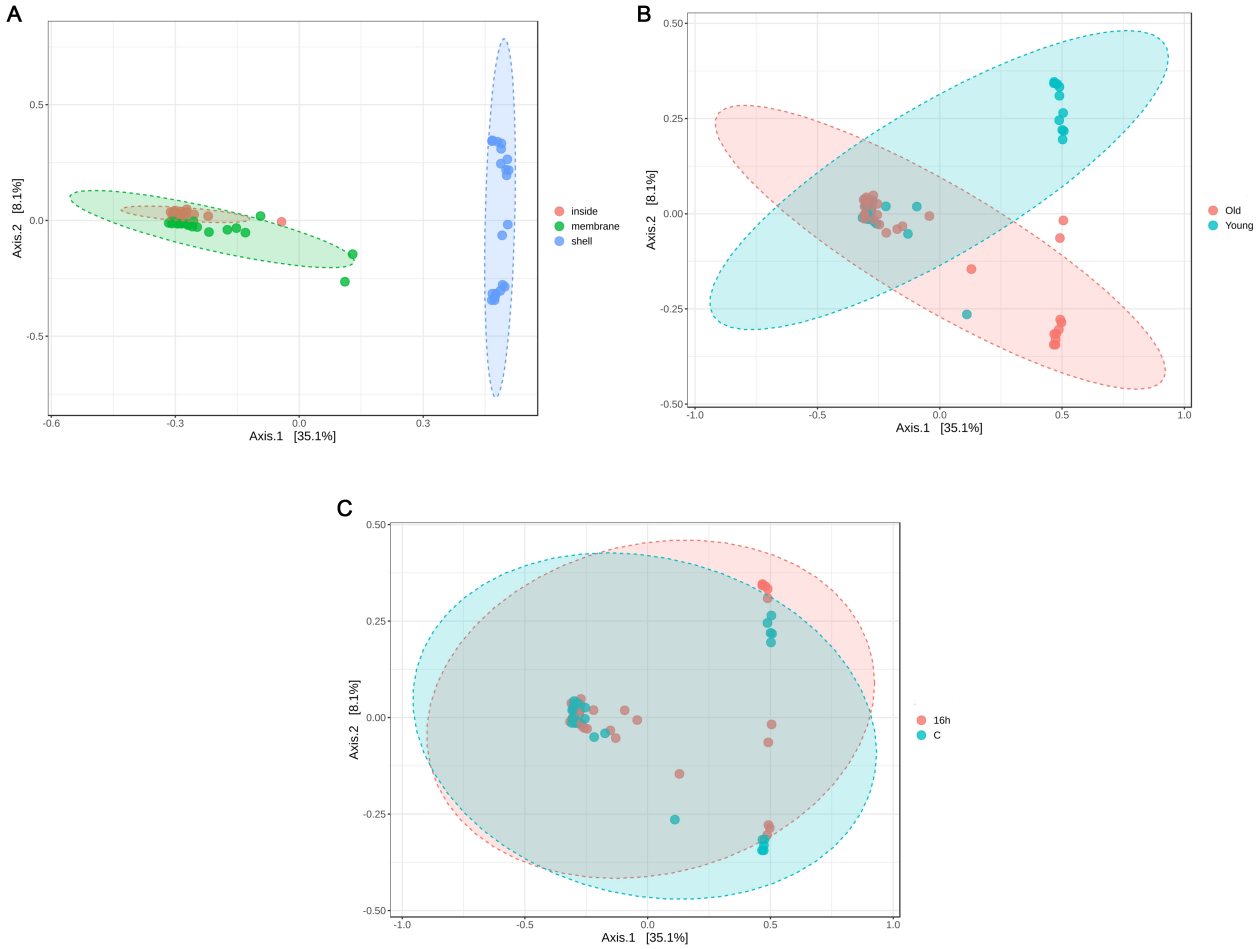
Venn diagrams showing the numbers of unique and shared OTUs and bacterial genera (numbers in parentheses) based on **(A)** egg parts, **(B)** age of breeder flocks, and **(C)** litter treatments.

Venn diagrams were constructed to analyze the common and unique OTUs and genera across the different egg parts (Figure 2). A total of 173 OTUs and 89 genera were shared among the three egg parts (eggshell, inner membrane, and egg content) (Figure 2A). The 10 most frequent genera within these shared groups were *Staphylococcus*, *Lactobacillus*, *Flexivirga*, *Salinicoccus*, *Pseudomonas*, *Rhodanobacter*, *Bacteroides*, *Paracoccus*, *Chujaibacter*, and *Rhodococcus*. Compared to the egg parts, litter treatments (Figure 2B) and the age of the flocks (Figure 2C) resulted in fewer unique OTUs and genera.

#### 3.3.6 Distribution of spoilage bacteria

The sum of relative abundances of eggshell spoilage-associated bacterial genera (*Alcaligenes, Enterococcus, Escherichia/Shigella, Proteus, Pseudomonas, Staphylococcus,* and *Streptococcus*) significantly increased after 16 h of litter treatment (Supplementary Table S4). Eggs from the younger flock contained a significantly higher ratio of these bacteria. The ratio of spoilage bacteria was highest on the eggshell surface, but interestingly, the difference between the egg membrane and egg content was also significant (Supplementary Table S4). This suggests some bacterial diffusion through the eggshell, although this could not be confirmed from the results of 16S rRNA sequencing.

## 4 Discussion

Floor eggs result in significant losses for broiler breeder farms and breeding companies due to their higher risk of bacterial contamination, which threatens both embryo viability and hatchery hygiene. If these eggs are excluded from incubation, the breeding farm incurs substantial losses. A floor egg ratio below 2% is considered good, and a range between 2 and 4% is generally acceptable. Floor egg ratios are typically higher in younger flocks. Cooper and Appleby (53) reported a 5% floor egg rate in 27–28-week-old broiler breeder hens, while other studies have recorded even higher percentages—up to 13.3% in 35-44-week-old flocks (54).

Although the hatchability of floor eggs is lower, the underlying causes are not yet fully understood. Two key, often interacting, factors have been identified: microbial infection of the embryo and ambient temperature (35). In our study, microbial contamination of the eggshell increased significantly, and hatchability decreased markedly after 16 h of contact with litter. While the number of culturable aerobic microbes increased on the surface of the floor eggs, no further increase was observed after 3 h. This finding indicates that time spent in the litter is not the sole determinant of aerobic bacterial load on the eggshell.

From a practical standpoint, this finding emphasizes the importance of frequent floor egg collection to preserve hatchability. We found no significant differences in coliform counts between litter treatments, and their numbers remained low across all samples. Our findings on microbial contamination are consistent with previous studies (1–3) and align with field observations. Peralta-Sánchez et al. (55) reported a negative relationship between aerobic mesophilic bacterial density on the eggshell and hatchability across 17 bird species. In contrast, other studies have reported no significant differences in hatchability between floor and nest eggs despite higher contamination levels in the former (4).

While the majority of studies on this topic focus on floor eggs, some have also investigated the effects of litter treatments (2, 3). In our study, hatchability decreased significantly only after 16 h of litter exposure, which correlated with increased embryonic mortality. Since aerobic bacterial counts on the eggshell plateaued after 3 h, the observed decline in hatchability was likely due to delayed egg cooling rather than microbial load. Prolonged exposure to warm litter delays temperature reduction, which is detrimental to embryo viability (53).

Interestingly, the number of aerobic bacteria on the eggshell decreased with hen age in our study, a result that contrasts with Moyle et al. (33), who reported increasing bacterial counts with hen age. However, their study was conducted on free-range laying hens, which are not directly comparable to our broiler breeder setup.

Egg quality is also influenced by hen age (56). As expected, the eggs of older hens (65–66 weeks) were heavier than those of younger hens (22–23 weeks), consistent with prior findings (26, 28, 57). Hatchability is generally higher in eggs from younger hens (3, 24, 27, 28, 30, 58), although some studies found no age-related differences (25) or even reported better hatchability in older hens (29). The discrepancies may be attributed to breed differences, flock age, or egg storage conditions before incubation (30). Optimal storage conditions must be tailored to the specific breeder flock to maximize embryo survival (30). These inconsistencies highlight the importance of standardizing hen age, breed, and storage conditions in hatchability studies.

Microbial diversity indices and OTU counts differed between egg components. Chao1 reflects species richness, Shannon accounts for both richness and evenness with sensitivity to rare OTUs, while Simpson emphasizes dominant OTUs (59). As expected, the eggshell exhibited the highest OTU counts and Chao1 diversity, followed by the membrane and then the egg content. Notably, the membrane had significantly higher OTU numbers and Chao1 indices than the egg content—a finding not previously reported, warranting further investigation.

The Shannon and Simpson indices also showed significant effects of litter treatment, although interactions among factors complicate interpretation. Our Shannon and Simpson values for egg content are consistent with those reported for egg whites in previous studies (40, 41).

Neither the membrane nor the egg content showed significant differences in OTU numbers or diversity indices across treatments, suggesting that the internal egg microbiota remain unaffected as long as the eggshell structure is intact.

Litter treatment significantly altered the microbial community composition on the eggshell at both the phylum and genus levels. The dominant phyla—*Firmicutes, Bacteroidetes, Actinobacteria,* and *Proteobacteria*—were consistent with earlier reports (5, 9, 60). In our study, litter exposure treatment increased the relative abundance of *Firmicutes* while reducing *Proteobacteria*. At the genus level, *Staphylococcus* abundance increased, and *Lactobacillus* decreased after 16 h of litter exposure. Older flocks had a lower abundance of *Staphylococcus* and *Lactobacillus* but a higher abundance of *Salinicoccus*. Although the litter microbiota was not characterized in our study, we attribute these differences to variations in litter microbial composition between flocks.

The eggshell microbiota originates from the hen’s reproductive and digestive tracts, as well as from the litter environment (6, 37). Litter is predominantly aerobic, which limits the survival of strictly anaerobic fecal bacteria. However, facultative anaerobes, aerotolerant, or spore-forming species can persist (12). While the majority of data on gut microbiota dynamics comes from broilers, some studies on laying phases indicate significant changes before laying and relative stability during the laying period (61, 62). *Firmicutes* dominate early laying phases (weeks 18–46), while *Bacteroidetes* increase during late phases (weeks 58–75) (34, 61). Litter microbiota composition is dynamic and influenced by factors such as diet, excreta, and litter moisture content.

Few studies have investigated temporal shifts in poultry manure microbiota (12, 63). Crippen et al. (13) and Zwirzitz et al. (14) reported that *Firmicutes* and *Actinobacteria* dominate mature litter, with *Actinobacteria* increasing over time—consistent with our eggshell microbiota results. Although eggshell microbiota has been studied (7, 60, 64, 65), the effect of flock or litter age on the eggshell microbial community has not been previously reported.

Neither litter treatment nor hen age significantly affected the microbial composition of the egg membrane. To the best of our knowledge, this is the first study to characterize the microbial composition of broiler hatching egg membranes. Dominant phyla included *Proteobacteria* and *Firmicutes*, with high abundances of *Actinobacteria, Cyanobacteria*, and *Bacteroidetes*. Minor phyla such as *Patescibacteria, Chlamydiae*, and *Planctomycetes* also exceeded 1% relative abundance. The genera *Pseudomonas* and *Flexivirga* together represented over 10% of the membrane microbiota. While *Pseudomonas* presence is expected, the detection of *Flexivirga*—a Gram-positive, aerobic, non-spore-forming genus in *Actinobacteria*—is novel in poultry-related research. *Flexivirga* has been previously isolated from soil (66), pine species (67), and mosquitoes (68), but not from poultry. Its relatively high abundance in the membrane (5.95%) and egg content (9.39%) compared to the eggshell (0.015%) suggests an oviduct origin. Previous studies have shown significant correlations between the microbial communities of the oviduct and egg white, as well as between the embryo gut and egg yolk microbiota (37, 41).

Our study examined the total egg content (yolk and white combined). Neither litter treatment nor breeder age significantly influenced its microbiome. Only a few studies have characterized yolk or white microbiota (37, 40, 41), but these findings generally indicate that *Proteobacteria, Firmicutes, Bacteroidetes,* and *Actinobacteria* are predominant. Despite analyzing the mixed content, our results are consistent, with *Proteobacteria* and *Firmicutes* as the dominant phyla, and minor phyla in similar proportions. *Flexivirga* was the most abundant genus in the egg content (9.4%), not previously reported in this context.

In litter-treated eggs, the relative abundance of spoilage-associated genera on the eggshell was nearly double that of untreated eggs. Although their abundance was lower internally, it was surprisingly higher in the membrane than in the egg content. This finding suggests that some spoilage organisms may penetrate the shell but remain below detection limits, even when sequencing-based methods are applied.

## 5 Conclusion

Litter treatment of hatching eggs increased the number of culturable aerobic microbes on the eggshell, with microbial penetration of the shell surface occurring after only 3 h. However, hatchability declined significantly only after 16 h of litter exposure. Since no bacterial translocation into the egg contents was detected, the reduced hatchability is likely due to prolonged exposure to uncontrolled temperatures, which may trigger premature embryonic development or compromise embryo viability. The primary cause of reduced hatchability was increased embryonic mortality. Litter treatment and flock age influenced only the composition of the eggshell microbiota; no significant effects were observed in the microbiota of the egg membrane or egg contents. The cumulative relative abundance of spoilage-associated bacterial genera—*Alcaligenes, Enterococcus, Escherichia/Shigella, Proteus, Pseudomonas, Staphylococcus, and Streptococcus*—increased significantly on the eggshell following litter exposure. Interestingly, the combined relative abundance of these genera was significantly higher in the egg membrane than in the egg content. A particularly novel finding was the high relative abundance of the genus *Flexivirga* in both the egg membrane and content, which has not been reported in previous studies. Further research is needed to clarify its origin, role, and potential impact on egg quality and embryonic development.

## Data Availability

The raw sequence data were deposited in the NCBI Sequence Read Archive (SRA) under BioProject ID PRJNA1012860.
