# Supplementary material for: Effects of litter exposure and flock age of broiler breeders on hatchability and the microbial composition of eggshells, egg membranes, and egg contents
Source: Front Vet Sci. 2025 Jun 26;12:1589607. doi: 10.3389/fvets.2025.1589607 (PMC12240755; doi:10.3389/fvets.2025.1589607)
Supplement: Supplementary file 1 [file Table_1.DOCX]

Table1S. Treatment effects on the relative abundances of bacterial genera of the eggshell (%) (n=10)

| eggshell | | | | | | | |
| --- | --- | --- | --- | --- | --- | --- | --- |
|  | age of breeders | | | mean (litter treatment) | BH-corrected p-value | | |
| genus | litter treatment | young | old |  | litter treatment | age of breeders | interaction |
| *Staphylococcus* | 0 h in litter | 16.36 | 1.70 | 9.03^b^ | 0.000 | 0.000 | 0.266 |
|  | 16 h in litter | 40.72 | 14.59 | 27.65^a^ |  |  |  |
|  | mean (age of breeders) | 28.54^a^ | 8.14^b^ |  |  |  |  |
| *Lactobacillus* | 0 h in litter | 22.26 | 15.06 | 18.66^a^ | 0.000 | 0.000 | 0.144 |
|  | 16 h in litter | 15.16 | 13.01 | 14.09^b^ |  |  |  |
|  | mean (age of breeders) | 18.71^a^ | 14.04^b^ |  |  |  |  |
| *Salinicoccus* | 0 h in litter | 3.83 | 21.30 | 12.56 ^a^ | 0.020 | 0.000 | 0.612 |
|  | 16 h in litter | 1.52 | 17.37 | 9.44 ^b^ |  |  |  |
|  | mean (age of breeders) | 2.67^b^ | 19.34^a^ |  |  |  |  |
| *Brachybacterium* | 0 h in litter | 2.77 | 7.42 | 5.09 ^a^ | 0.015 | 0.000 | 0.702 |
|  | 16 h in litter | 1.66 | 6.42 | 4.04 ^b^ |  |  |  |
|  | mean (age of breeders) | 2,21^b^ | 6.92^a^ |  |  |  |  |
| *Bacteroides* | 0 h in litter | 6.00 | 4.40 | 5.20 | 0.310 | 0.187 | 0.802 |
|  | 16 h in litter | 5.00 | 3.80 | 4.40 |  |  |  |
|  | mean (age of breeders) | 5.50 | 4.10 |  |  |  |  |
| *Romboutsia* | 0 h in litter | 3.00 | 2.00 | 2.50 | 0.885 | 0.757 | 0.061 |
|  | 16 h in litter | 2.40 | 3.20 | 2.80 |  |  |  |
|  | mean (age of breeders) | 2.70 | 2.60 |  |  |  |  |
| *Brevibacterium* | 0 h in litter | 1.60 | 4.40 | 3.00 | 0.573 | 0.000 | 0.261 |
|  | 16 h in litter | 1.00 | 4.40 | 2.70 |  |  |  |
|  | mean (age of breeders) | 1.30^b^ | 2.55^a^ |  |  |  |  |
| *Ruminococcus_ torques_group* | 0 h in litter | 3.60 | 1.20 | 2.40^b^ | 0.044 | 0.000 | 0.447 |
|  | 16 h in litter | 2.60 | 0.80 | 1.70^a^ |  |  |  |
|  | mean (age of breeders) | 3.10^a^ | 1.00^b^ |  |  |  |  |
| *Turicibacter* | 0 h in litter | 1.45 | 1.36 | 1.40^a^ | 0.001 | 0.137 | 0.044 |
|  | 16 h in litter | 1.05 | 1.39 | 1.22^b^ |  |  |  |
|  | mean (age of breeders) | 1.25 | 1.38 |  |  |  |  |
| *Enterococcus* | 0 h in litter | 2.40 | 2.20 | 2.23^a^ | 0.001 | 0.735 | 0.301 |
|  | 16 h in litter | 1.00 | 1.60 | 1.30^b^ |  |  |  |
|  | mean (age of breeders) | 1.70 | 1.90 |  |  |  |  |
| *Jeotgalicoccus* | 0 h in litter | 3.24 | 0.79 | 2.02 |  |  |  |
|  | 16 h in litter | 2.30 | 1.70 | 2.01 | 0.543 | 0.016 | 0.061 |
|  | mean (age of breeders) | 2.77^a^ | 1.25^b^ |  |  |  |  |
| *Corynebacterium* | 0 h in letter | 2.20 | 0.20 | 1.20 |  |  |  |
|  | 16 h in litter | 1.60 | 0.40 | 1.00 | 0.310 | 0.000 | 0.266 |
|  | mean (age of  breaders) | 1.90 | 3.00 |  |  |  |  |
| *Yaniella* | 0 h in litter | 1.99 | 1.59 | 1.79 | 0.105 | 0.039 | 0.003 |
|  | 16 h in litter | 1.34 | 3.17 | 2.25 |  |  |  |
|  | mean (age of breeders) | 1.67^b^ | 2.38^a^ |  |  |  |  |

The table contains only genera above 1% relative abundance; ^a,b^ means with different superscripts of the main treatment averages are significantly different (p<0.05).

Table 2S. Treatment effects on the relative abundances of bacterial genera of the egg membrane (%) (n=10)

| membrane | | | | | | | |
| --- | --- | --- | --- | --- | --- | --- | --- |
|  | age of breeders | | | mean (litter treatment) | BH-corrected p-value | | |
| genus | litter treatment | young | old |  | litter treatment | age of breeders | interaction |
| *Pseudomonas* | 0 h in litter | 9.27 | 5.35 | 7.31 | 0.806 | 0.806 | 0.806 |
|  | 16 h in litter | 7.53 | 8.07 | 7.80 |  |  |  |
|  | mean (age of breeders) | 8.40 | 6.71 |  |  |  |  |
| *Flexivirga* | 0 h in litter | 6.47 | 2.78 | 4.63 | 0.806 | 0.806 | 0.806 |
|  | 16 h in litter | 4.11 | 3.68 | 3.90 |  |  |  |
|  | mean (age of breeders) | 5.29 | 3.23 |  |  |  |  |
| *Staphylococcus* | 0 h in litter | 1.53 | 4.45 | 2.99 | 0.806 | 0.806 | 0.806 |
|  | 16 h in litter | 2.02 | 6.18 | 4.10 |  |  |  |
|  | mean (age of breeders) | 1.77 | 5.31 |  |  |  |  |
| *Paracoccus* | 0 h in litter | 1.94 | 1.17 | 1.56 | 0.806 | 0.806 | 0.806 |
|  | 16 h in litter | 1.25 | 2.13 | 1.69 |  |  |  |
|  | mean (age of breeders) | 1.59 | 1.65 |  |  |  |  |
| *Rhodanobacter* | 0 h in litter | 2.21 | 1.42 | 1.82 | 1.000 | 0.806 | 0.806 |
|  | 16 h in litter | 2.38 | 1.37 | 1.88 |  |  |  |
|  | mean (age of breeders) | 2.30 | 1.40 |  |  |  |  |
| *Enhydrobacter* | 0 h in litter | 0.65 | 0.58 | 0.62 | 0.806 | 0.806 | 0.806 |
|  | 16 h in litter | 1.61 | 3.18 | 2.40 |  |  |  |
|  | mean (age of breeders) | 1.13 | 1.88 |  |  |  |  |
| *Candidimonas* | 0 h in litter | 1.39 | 1.60 | 1.49 | 0.806 | 0.844 | 0.806 |
|  | 16 h in litter | 1.05 | 0.93 | 0.99 |  |  |  |
|  | mean (age of breeders) | 1.22 | 1.26 |  |  |  |  |
| *Chujaibacter* | 0 h in litter | 1.22 | 0.94 | 1.08 | 0.806 | 0.806 | 0.806 |
|  | 16 h in litter | 1.27 | 1.10 | 1.19 |  |  |  |
|  | mean (age of breeders) | 1.25 | 1.02 |  |  |  |  |
| *Ruminococcaceae_ NK4A214_group* | 0 h in litter | 1.35 | 1.38 | 1.36 | 0.806 | 0.974 | 0.806 |
|  | 16 h in litter | 0.93 | 0.55 | 0.74 |  |  |  |
|  | mean (age of breeders) | 1.14 | 0.97 |  |  |  |  |
| *Salinicoccus* | 0 h in litter | 1.91 | 0.21 | 1.06 | 0.989 | 0.806 | 0.806 |
|  | 16 h in litter | 0.36 | 1.81 | 1.08 |  |  |  |
|  | mean (age of breeders) | 1.13 | 1.01 |  |  |  |  |
| *Mycobacterium* | 0 h in litter | 1.14 | 1.32 | 1.23 | 0.806 | 0.806 | 0.806 |
|  | 16 h in litter | 0.77 | 1.47 | 1.12 |  |  |  |
|  | mean (age of breeders) | 0.95 | 1.40 |  |  |  |  |
| *Allorhizobium* | 0 h in litter | 1.08 | 0.64 | 0.86 | 0.806 | 0.806 | 0.806 |
|  | 16 h in litter | 1.22 | 1.27 | 1.24 |  |  |  |
|  | mean (age of breeders) | 1.15 | 0.96 |  |  |  |  |
| *Rhodococcus* | 0 h in litter | 0.80 | 2.40 | 1.60 | 1.000 | 0.806 | 0.806 |
|  | 16 h in litter | 1.80 | 1.00 | 1.40 |  |  |  |
|  | mean (age of breeders) | 1.30 | 1.70 |  |  |  |  |
| *Rothia* | 0 h in litter | 0.01 | 3.95 | 1.98 | 0.806 | 0.806 | 0.806 |
|  | 16 h in litter | 0.06 | 0.00 | 0.03 |  |  |  |
|  | mean (age of breeders) | 0.03 | 1.97 |  |  |  |  |
| *Lactobacillus* | 0 h in litter | 1,32 | 0.17 | 0.74 | 0.806 | 0.806 | 0.806 |
|  | 16 h in litter | 2.11 | 1.15 | 1.63 |  |  |  |
|  | mean (age of breeders) | 1.72 | 0.66 |  |  |  |  |

The table contains only genera above 1% relative abundance

Table 3S. Treatment effects on the relative abundances of bacterial genera of the egg content (%) (n=10)

| egg content | | | | | | | |
| --- | --- | --- | --- | --- | --- | --- | --- |
|  | age of breeders | | | mean (litter tratment) | BH-corrected p-value | | |
| genus | litter treatment | young | old |  | litter treatment | age of breeders | interaction |
| *Flexivirga* | 0 h in litter | 6.41 | 7.68 | 7.04 | 0.716 | 0.965 | 0.965 |
|  | 16 h in litter | 6.09 | 6.17 | 6.13 |  |  |  |
|  | mean (age of breeders) | 6.25 | 6.92 |  |  |  |  |
| *Rhodanobacter* | 0 h in litter | 4.39 | 2.44 | 3.42 | 0.716 | 0.716 | 0.799 |
|  | 16 h in litter | 4.94 | 2.51 | 3.72 |  |  |  |
|  | mean (age of breeders) | 4.67 | 2.47 |  |  |  |  |
| *Paracoccus* | 0 h in litter | 1.98 | 0.86 | 1.42 | 0.716 | 0.716 | 0.716 |
|  | 16 h in litter | 5.63 | 0.86 | 3.25 |  |  |  |
|  | mean (age of breeders) | 3.81 | 0.86 |  |  |  |  |
| *Rhodococcus* | 0 h in litter | 2.00 | 2.80 | 2.40 | 0.965 | 0.941 | 0.941 |
|  | 16 h in litter | 2.40 | 2.40 | 2.41 |  |  |  |
|  | mean (age of breeders) | 2.20 | 2.60 |  |  |  |  |
| *Chujaibacter* | 0 h in litter | 1.64 | 2.39 | 2.01 | 0.716 | 0.965 | 0.941 |
|  | 16 h in litter | 1.83 | 1.34 | 1.59 |  |  |  |
|  | mean (age of breeders) | 1.73 | 1.87 |  |  |  |  |
| *Alkanibacter* | 0 h in litter | 1.48 | 1.68 | 1.58 | 0.965 | 0.970 | 0.965 |
|  | 16 h in litter | 1.83 | 1.57 | 1.70 |  |  |  |
|  | mean (age of breeders) | 1.65 | 1.62 |  |  |  |  |
| *Mycobacterium* | 0 h in litter | 1.50 | 2.55 | 2.03 | 0.716 | 0.716 | 0.716 |
|  | 16 h in litter | 1.59 | 1.65 | 1.62 |  |  |  |
|  | mean (age of breeders) | 1.55 | 2.10 |  |  |  |  |
| *Ruminococcaceae NK4A214_group* | 0 h in litter | 0.88 | 1.20 | 1.04 | 0.716 | 0.716 | 0.965 |
|  | 16 h in litter | 1.51 | 2.36 | 1.93 |  |  |  |
|  | mean (age of breeders) | 1.20 | 1.78 |  |  |  |  |
| *Enhydrobacter* | 0 h in litter | 2.71 | 3.01 | 2.86 | 0.716 | 0.970 | 0.716 |
|  | 16 h in litter | 1.91 | 0.50 | 1.20 |  |  |  |
|  | mean (age of breeders) | 2.31 | 1.75 |  |  |  |  |
| *Lactobacillus* | 0 h in litter | 0.05 | 0.00 | 0.03 | 0.716 | 0.716 | 0.716 |
|  | 16 h in litter | 0.28 | 4.92 | 2.60 |  |  |  |
|  | mean (age of breeders) | 0.16 | 2.47 |  |  |  |  |
| *Streptococcus* | 0 h in litter | 0.40 | 0.00 | 0.20 | 0.716 | 0.716 | 0.716 |
|  | 16 h in litter | 0.40 | 5.00 | 2.70 |  |  |  |
|  | mean (age of breeders) | 0.40 | 2.50 |  |  |  |  |
| *Cloacibacterium* | 0 h in litter | 0.79 | 2.85 | 1.82 | 0.96 5 | 1.000 | 0.965 |
|  | 16 h in litter | 1.05 | 0.13 | 0.59 |  |  |  |
|  | mean (age of breeders) | 0.92 | 1.49 |  |  |  |  |
| *Methylobacterium* | 0 h in litter | 0.83 | 1.10 | 0.97 | 0.965 | 0.965 | 0.965 |
|  | 16 h in litter | 1.41 | 1.57 | 1.49 |  |  |  |
|  | mean (age of breeders) | 1.12 | 1.33 |  |  |  |  |

The table contains only genera above 1% relative abundance

Table 4S. Treatment effects on the relative abundances of egg spoilage causing bacterial genera (n=10)

| litter treatment | age of breeders | part of the egg | relative abundances of spoilage bacteria |
| --- | --- | --- | --- |
| 0 hour | old | shell | 0.0494 |
|  |  | membrane | 0.1149 |
|  |  | content | 0.0167 |
| 0 hour | young | shell | 0.1989 |
|  |  | membrane | 0.1176 |
|  |  | content | 0.0259 |
| 16 hours | old | shell | 0.1717 |
|  |  | membrane | 0.1486 |
|  |  | content | 0.0649 |
| 16 hours | young | shell | 0.4301 |
|  |  | membrane | 0.1059 |
|  |  | content | 0.0285 |
| *litter treatment* | | | |
| 0 hour |  |  | 0.0872^b^ |
| 16 hours |  |  | 0.1583^a^ |
| SEM |  |  | 0.0120 |
| *age of breeders* | | | |
| old |  |  | 0.0943^b^ |
| young |  |  | 0.1511^a^ |
| SEM |  |  | 0.0120 |
| *part of the egg* | | | |
| shell |  |  | 0.2125^a^ |
| membrane |  |  | 0.1217^b^ |
| content |  |  | 0.0340^c^ |
| SEM |  |  | 0.0140 |
| *p*-values |  |  |  |
| litter treatment |  |  | 0.000 |
| age of breeders |  |  | 0.001 |
| part of the egg |  |  | 0.000 |
| litter x age |  |  | 0.000 |
| litter x egg part |  |  | 0.858 |
| age x egg part |  |  | 0.000 |
| litter x age x egg part |  |  | 0.099 |

^a,b^ means with different superscripts of the main treatment averages are significantly different (p<0.05).
